# Pinocembrin-7-Glucoside (P7G) Reduced Postharvest Blue Mold of Navel Orange by Suppressing *Penicillium italicum* Growth

**DOI:** 10.3390/microorganisms8040536

**Published:** 2020-04-08

**Authors:** Chuying Chen, Jinyin Chen, Chunpeng Wan

**Affiliations:** 1 Jiangxi Key Laboratory for Postharvest Technology and Non-destructive Testing of Fruits & Vegetables/Collaborative Innovation Center of Postharvest Key Technology and Quality Safety of Fruit and Vegetables, College of Agronomy, Jiangxi Agricultural University, Nanchang 330045, China; cy.chen@jxau.edu.cn; 2 Pingxiang University, Pingxiang 337055, China

**Keywords:** pinocembrin-7-glucoside, blue mold, navel orange, *Penicillium italicum*, antifungal activity

## Abstract

The current study aimed to examine the in vitro and in vivo antifungal potential of pinocembrin-7-glucoside (P7G). P7G is an antifungal flavanone glycoside isolated from *Ficus hirta* Vahl. fruit against *Penicillium italicum*, a causative pathogen of blue mold disease in citrus fruit, and this study elucidates its possible action mechanism. P7G had a prominent mycelial growth inhibitory activity against *P. italicum*, with an observed half maximal effective concentration, minimum inhibitory concentration and minimum fungicidal concentration of 0.08, 0.2, and 0.8 g/L, respectively. The data from the in vivo test show that P7G significantly reduced blue mold symptoms and disease development of *P. italicum* in artificially inoculated “Newhall” navel orange. Compared to the control, increases in the cell membrane permeability of *P. italicum* supernatant and decreases in the intracellular constituent (e.g., soluble protein, reducing sugar, and total lipid) contents of *P. italicum* mycelia were identified, supporting scanning electron microscopy and transmission electron microscopy observations. Furthermore, a marked decline in both chitin and glucanase contents of *P. italicum* mycelia treated with P7G was induced by increasing its related degrading enzyme activities, suggesting that the cell wall structure was destroyed. The current study indicated that P7G may be a novel alternative for reducing blue mold by suppressing mycelial growth of *P. italicum* via a cell membrane/wall-targeting mechanism.

## 1. Introduction

By far, China is the largest country in the world in terms of both citrus yield and cultivated area, and it has a large variety of species and varieties, such as loose-skin mandarin, sweet orange, pummelo, kumquat, lemon, grapefruit, citron, and different special hybrids [1,2]. The postharvest nutritional quality and storage life of citrus fruit were limited by a variety of fungal diseases, including citrus blue mold (*Penicillium italicum*), green mold (*Penicillium digitatum*), sour rot (*Geotrichum citri-aurantii*), brown spot (*Alternaria alternata*), stem-end rot (*Diaporthe citri*), and black spot (*Phyllosticta citricarpa*) [3,4,5,6,7]. Among them, blue mold is the primary postharvest disease that causes 20–50% of citrus fruit loss in China during postharvest disposal [3]. In past decades, some commercial fungicides such as imazalil, prochloraz, or thiabendazole have been the primary means of controlling these diseases [8,9,10]. However, increasing public concerns regarding chemical residues on human health, environmental pollution, and resistance pathogens due to excessive use of chemical fungicides have initiated an investigation into alternative strategies for reducing postharvest fungal decay without any human, environmental, or plant toxicity [11,12,13]. Recently, a huge number of studies have documented the antifungal effects of plant-derived natural compounds, generally regarded as safe (GRAS) substances, for controlling postharvest blue mold caused by *P. italicum* incurring serious deterioration of citrus fruit.

*Ficus hirta* Vahl. (family of Moraceae) fruits, which are also called “**wǔ zhǐ Máo Táo guǒ**”, are used as medicine and edible food for the treatment of constipation, inflammation, postpartum hypogalactia, tumors, and cancers by Hakka people in Southern China [14,15,16,17]. In our previous study, we reported that the ethanol extracts from *F. hirta* Vahl. fruits displayed prominent antifungal activity against *P. italicum*, *P. digitatum*, *A. citri*, *G. citri-aurantii*, and other pathogens in vitro [18]. Particularly, the ethanol extract of *F. hirta* Vahl. fruits was found to be able to control postharvest blue mold decay and to improve the preservation effects of “Newhall” navel oranges, “97-2” Nangfeng mandarins and “Xinyu” tangerines [19,20,21]. Correspondingly, the extract of *F. hirta* Vahl. fruits might be developed into a natural alternative to the traditional fungicides for controlling postharvest fungal diseases of horticultural crops.

**Figure 1:**
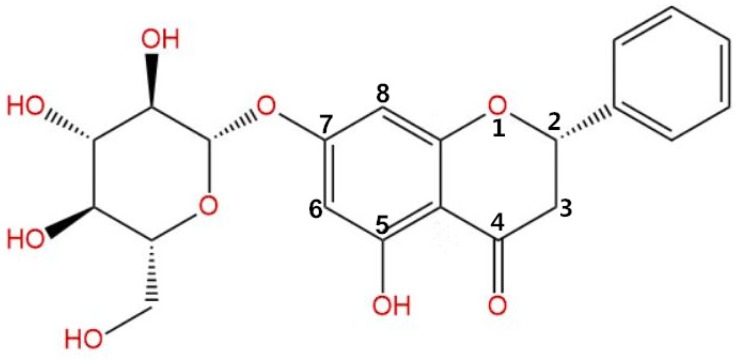
Chemical structure of pinocembrin-7-glucoside (P7G).

Flavonoid glycosides are a group of flavonoids mainly isolated from some plant tissues, such as *Astragalus membranaceus* leaves, *Ficus hirta* Vahl. fruits, *Lilium lancifolium* flowers, poplar buds, and *Plinia cauliflora* leaves [22,23,24,25,26], and are known for their potent antifungal activity, which occurs through a lipid peroxidation mechanism. In our previous research, pinocembrin-7-glucoside (P7G; Figure 1) or pinocembroside, one of the flavonoid glycosides, was isolated as one of the major antifungal compounds in *Ficus hirta* Vahl. fruits [23]. Recently, P7G has become the focus of increased attention due to its strong preventive effects against hepatic steatosis and its prominent protective effects against ethanol-induced liver injury [26,27]. A previous study which bore out the antifungal activity of P7G on *P. italicum* in potato dextrose agar (PDA) media showed that P7G was successful at inhibiting both *Penicillium* spp. mycelial growth [23,28,29].

However, to date, there have been no reports on the antifungal activity and possible action mechanisms of P7G on postharvest pathogenic fungus in citrus fruit, especially those concerning *P. italicum*, which infects fresh citrus fruit. Therefore, the present study aimed to evaluate the antifungal activity of P7G against *P.* *italicum* both in vitro and in vivo tests. Moreover, focus on the possible mechanisms involving morphological changes, intracellular constituent losses and plasma membrane permeability in *P. italicum* were also studied.

## 2. Materials and Methods

### 2.1. Fruit Material

Navel oranges (*Citrus sinensis* L., Osbeck cv. Newhall) were hand-harvested by the end of November 2017 at a local orchard in Anyuan County, situated in the Southeast of Ganzhou city (Jiangxi Province), China. The physiologically mature fruits were chosen by keeping in mind the health and size (240–280 g) of the fruit, identical color (citrus color index: CCI, 5.8–7.0), and the lack of any bruises and disease symptoms.

### 2.2. Phytopathogenic Fungi Isolation

The phytopathogenic fungi of *P. italicum P. digitatum*, *G. citri-aurantii*, *A. citri*, *D. citri*, and *Colletotrichum gloeosporioides* were taken from the rotted citrus fruits with representative disease symptoms of blue mold, green mold, sour rot, black rot, stem-end rot, and anthracnose, and were properly identified by using DNA sequencing done by the National Center for Industrial Culture Collection (CICC) of China. The pure fungi were expanded on a PDA medium at 25 ± 1 °C. On day seven of culturing, the *P.* *italicum* spore suspension was collected, filtered, and set at the optimum level of 1.0 × 10^6^ CFU/mL using a Countess-II FL automatic cell counter.

### 2.3. P7G Isolation and Purification

The P7G with 92.57% purity used in the current study, originally isolated from *F. hirta* Vahl. fruits [23], was suspended in 80% ethanol to obtain a stock solution of 10 g/L.

### 2.4. In Vitro Antifungal Activity Assay

#### 2.4.1. Minimal Inhibitory Concentration (MIC) and Minimum Fungicidal Concentration (MFC)

Both minimal inhibitory concentration (MIC) and minimum fungicidal concentration (MFC) were evaluated following our previous method reported by Chen et al. [29], where the MIC and MFC values were considered as the lowest concentration of P7G that completely inhibited mycelial growth of six tested phytopathogenic fungi after incubation for 2 and 6 days, respectively.

#### 2.4.2. Assay of P7G on Mycelial Growth of *P. italicum*

The antifungal properties of P7G on mycelial growth of *P. italicum* were identified according to our previously described method [3,28]. In short, the stock solution of P7G was further diluted using an optimum quantity of 0.5% sterile Tween 80, and then mixed up in PDA to make a range of dilutions (0, 0.025, 0.05, 0.1, 0.2, 0.4, and 0.8 g/L). Afterward, a 5-mm-diameter mycelial disk, cut from the periphery of 7-day-old culture of *P. italicum*, was dropped on the center of each PDA dish. After incubation at 25 °C in the dark for 7 days, the colony diameters of these dishes were measured, and the inhibitory rate of *P. italicum* growth was calculated according to the formula: (the mean colony diameter of control set—the mean colony diameter of P7G-treated set)/ the mean colony diameter of control set × 100. Each treatment and control group were replicated four times, and the experiment was carried out separately twice. The half maximal effective concentration was expressed as EC_50_ (with the P7G-treated concentration effectively inhibiting growth rate by 50%) and calculated based on the linear regression of the mycelial growth inhibition (MGI) on the log-transformed P7G concentrations [29].

#### 2.4.3. Assay of P7G on Spore Germination of *P. italicum*

The antifungal stability of P7G was determined by the spore germination assay according to the method we reported in a previous study [3]. Briefly, the tested *P. italicum* strain was grown on PDA plate medium at 25 °C for 7days. P7G was prepared by dissolving moderate amounts in sterile 0.5% Tween 80, and individually diluted with potato dextrose broth (PDB) to obtain a series of concentrations (0, 0.025, 0.05, 0.1, 0.2, 0.4, and 0.8 g/L). Afterward, 5 μL of *P. italicum* spore suspension was added into both the control (0-concentrate) and P7G-treated slides. After incubation at 25 °C in the dark for 14 h, approximately 100 spores per replicate were microscopically observed, and the germination inhibitory rate of *P. italicum* spore was calculated according to the formula: (the mean amount of germinated spore in the control slide—the mean amount of germinated spore in the P7G-treated slide)/ the mean amount of germinated spore in the control slide × 100. Each treatment and control group was replicated four times, and the experiment was carried out separately twice.

### 2.5. In Vivo Antifungal Activity Assay on Navel Orange Fruit

The effects of P7G on lesion diameter and disease severity in navel oranges were determined following the method previously described with slight modifications [30,31]. The sterilized navel oranges were wounded (4 mm diameter and 2-mm deep) using a sterile puncher at the equatorial side on each fruit, and then divided in five randomized groups for various P7G treatment levels (0, 1, 2, 4, and 8 g/L). About 20 μL of solutions either containing P7G or not (the control) were injected into each wound on the citrus fruit. After air-drying for 30 min, an equal volume of fungal spore suspension (5 × 10^4^ CFU/mL) was also injected in each respective wound of the citrus fruit. All fruits were then put in containers (45 × 36 cm polyethylene-lined plastic frames) which maintained higher relative humidity levels (90–95%) at 25 °C. The lesion diameter and disease severity of the P7G-treated and the control fruits’ wounds were observed at 3, 5, and 7 days post-inoculation. Three replicates of 30 fruits were used per treatment, and the experiment was performed twice. The lesion diameter was measured by the cross method using a Vernier caliper. The disease severity was evaluated and expressed as a percentage following a previously described method [30].

### 2.6. Microscopic Observations

The effects of P7G on morphologic and ultra-structure changes of *P. italicum* were observed using both scanning electron microscopy (SEM) and transmission electron microscopy (TEM). SEM (JSM-6360LV, JEOL Ltd., Tokyo, Japan) and TEM (JEM-1400, JEOL Ltd., Tokyo, Japan) were used to observe the *P. italicum* mycelia treated with P7G at different concentrations (0, EC_50_, and MIC), following the methods previously reported by Tao et al. [32] with some modifications.

For SEM preparation, about 1.0 g of mycelia were collected from P7G-treated and control PDB medium and promptly fixed with 0.1 M phosphate-buffered saline (PBS; pH 7.2) containing 3.0% (*v*/*v*) glutaraldehyde at 4 °C for 8 h. To remove glutaraldehyde, the fixed mycelia was washed five times with 0.1 M PBS (pH 7.2) followed by gradient ethanol elution (30%, 50%, 70%, 80%, 90%, and 100%, *v*/*v*), for 15 min in each ethanol dilution. The specimens were immersed in an iso-amyl acetate solution for 20 min, and finally dried in a critical point drier (CPD; Samdri-795, Tousimis Co., LTD, Rockville, America). Following gold-coating, the prepared samples were observed by SEM operating at a 1500 × magnification.

For TEM preparation, the dehydrated samples were passed through the solution of epoxy propane and epoxy resin (1:1) for 2 h, and then embedded in pure epoxy media for 12 h. Following polymerization at 37 °C for 12 h, 45 °C for 12 h, and 60 °C for 48 h, the prepared specimens were double-stained with 3% (*m*/*v*) uranyl acetate and lead citrate, and finally observed by TEM operating at a 15000 × magnification.

### 2.7. Determination of Cell Membrane Permeability

The extracellular conductivity (EC) of *P. italicum* mycelia was determined using an electrical conductivity meter (ST3100c/F, Ohaus Co., Ltd., New Jersey, America) following P7G treatment by a previous method with minor alterations [3]. The extracellular conductivity was observed at 0, 1, 2, 4 and 6 h for the P7G-treated and the control groups, and results showed extracellular conductivity (μs/cm).

The rate of cell lysis rate in the P7G-treated and control groups was examined using a spectrophotometer. The level of cell lysis was explained as the difference between the initial and final absorbance recorded at 650 nm, and the calculations were made using the following formula:

$$\mathrm{Cell}\;\mathrm{lysis}\;\mathrm{rate}\;(\mathrm{CLR},\;\%)=\frac{\mathrm{At}-A0}{A0}\times 100$$

where At and A0 are the absorbance determined for the P7G-treated and control PDB groups, respectively.

The nucleic acid leakage from *P. italicum* mycelia due to the P7G treatment was detected following the method given by Huang and colleagues [33], with slight modifications. The absorbance at 260 nm was examined at 0, 1, 2, 4 and 6 h, and the leakage of nucleic acids from the P7G-treated and control group supernatants was expressed as OD_260_.

### 2.8. Measurement of Intracellular Constituent Contents

#### 2.8.1. Mycelial Growth Weight

The effect of P7G on the mycelial growth weight of *P. italicum* was evaluated according to the method described previously by Chen et al. [29], with minor modifications, and the data were expressed as the mycelial volume (dry weight) based on 100 mL PDA (g/100mL).

#### 2.8.2. Evaluation of Soluble Protein and Reducing Sugar Levels

The contents of soluble protein and reducing sugar in *P. italicum* mycelia treated with P7G at various concentrations (0, EC_50_ and MIC) were determined using the method described by Chen et al. [3] with minor modifications, and were measured to calculate the soluble protein and reducing sugar contents (mg/g) using the standards of bovine serum albumin and glucose, respectively.

#### 2.8.3. Total Lipid Content

The total lipid content of *P. italicum* mycelia with P7G at various concentrations (0, EC_50_, and MIC) was identified using the method as described by Tao et al. [32], and expressed as milligrams per gram (mg/g) of frozen weight.

### 2.9. Assays of Cell Wall Components Content and Enzyme Activities

The contents of cell wall components in *P. italicum* mycelia treated with P7G at 0, EC_50_, and MIC were determined using the method described by Stalhberger et al. [34], with minor modifications. The chitin content was measured using D-glucosamine sulfate as a standard, and the glucanase content was calculated as the sum of alkali soluble and insoluble sugar contents and was expressed as milligrams per gram (mg/g).

The activities of chitinase (CHI) and β-1,3-glucanase (β-Glu) were determined by our previously described method [30]. One unit of the above two activities was equal to the amount of enzyme that catalyzed the production of 1.0 nM N-acetyl-d-glucosamine (GlcNAc) and 1 μg of reducing sugar per min of 1 g fresh weight mycelia. Both CHI and β-Glu activities were expressed as U/g of frozen weight.

### 2.10. Statistical Analysis

All experimental data are reported as the means ± standard error (S.E.) and were analyzed using SPSS version 17.0 (SPSS Inc., Chicago, IL, USA). Data from extracellular conductivity, cell lysis rate, nucleic acid leakage, mycelial growth weight, soluble protein content, reducing sugar content, cell wall component contents, and related enzyme activities assays were analyzed using Duncan’s test, and significant differences were observed by applying one-way analysis of variance at the 5% level.

## 3. Results

### 3.1. P7G Displays Potential Used as Antifungal Agent in Citrus Phytopathogenic Fungi

Based on the observations of six tested phytopathogenic fungi growth on PDA medium containing various concentrations (0, 0.025, 0.05, 0.1, 0.2, 0.4, and 0.8 g/L) of P7G during incubation at 25 °C, P7G had strong inhibitory effect as MIC and MFC against six tested phytopathogenic fungi in citrus fruit. As shown in Table 1, the values of MIC and MFC of P7G against the tested fungi of *A. citri*, *C. gloeosporioides*, *D. citri*, *G. citri-aurantii*, *P. digitatum*, and *P. italicum* were found in the range of 0.1 to 0.8 g/L. In this assay, *Diaporthe citri* was found as the most susceptible fungus to P7G with the lowest MIC value (0.1 g/L). Among these phytopathogenic fungi, *A. citri* showed the least susceptibility to P7G with the highest MIC and MFC value (0.8 g/L). These results are in line with our previous study of clove essential oil (CEO) [35], and indicating that the in vitro antifungal efficiency of CEO may be similar and better than traditional citrus preservatives, with the values of MIC against *P. italicum*, *P. digitatum*, *G. citri-aurantii*, and *A. citri* ranging from 0.2 to 1.0 mL/L. Peng et al. [36] also reported that pinocembrin had potent antifungal activity against *P. italicum*, with MIC of 0.4 g/L. Our results suggested that P7G, as a flavonone derived from *F. hirta* Vahl. fruits, could be used as a novel antifungal agent to control citrus postharvest diseases.

### 3.2. P7G has Antifungal Activity Against P. italicum

**Figure 2:**
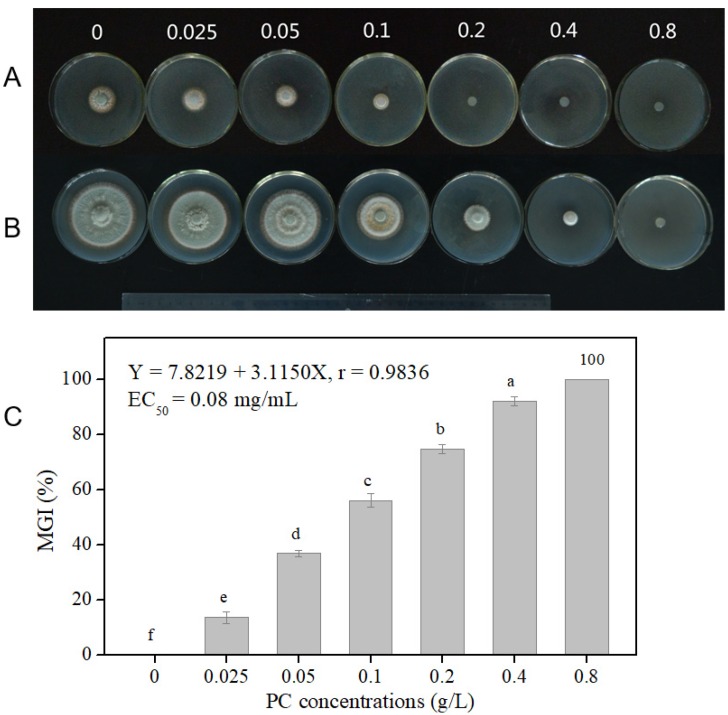
The minimal inhibitory concentration (MIC), the minimum fungicidal concentration (MFC) and the inhibitory effect of P7G against *P. italicum* on the second day (**A**), sixth day (**B**), and seventh day (**C**) after inoculation. Different letters (a–f) at each interval in Figure 1C indicate significant differences at *p* < 0.05 according to Duncan’s test.

As shown in Table 1 and Figure 2, the 0.2 and 0.8 g/L P7G treatments completely inhibited *P. italicum* mycelial growth on the second and sixth days of incubation, respectively. Therefore, the values of MIC and MFC in response to P7G against *P. italicum* were 0.2 and 0.8 g/L, respectively.

As shown in Figure 2C, a significant inhibitory effect of P7G on *P. italicum* mycelial growth on PDA medium was seen in a dose-dependent fashion where the higher P7G concentration gave higher MGI. P7G at 0.2 g/L could significantly suppress the mycelial growth of *P. italicum*, being inhibited by 74.7% compared with the control group (Figure 2C, *p* < 0.05), which was twice that of the MGI seen at 0.025 g/L (36.9%). Particularly, a complete inhibition of *P. italicum* mycelial growth was achieved at 0.8 g/L of P7G. Furthermore, by calculating the MGI under different P7G concentrations, a linear regression of the MGI of *P. italicum* (Y) on the log-transformed P7G concentrations (X) was determined—Y = 7.8219 + 3.1150X, r = 0.9836 (Figure 2C)—with the half maximal effective concentration (EC_50_) of P7G against *P. italicum* being 0.08 g/L.

The spore germination of *P. italicum* markedly decreased with increasing P7G-treated concentrations (*p* < 0.05; Table 2). After incubation in PDB for 14 h, the inhibitory rate of *P. italicum* spore decreased by more 50% in the presence of 0.05 g/L P7G. When the P7G-treated concentration reached 0.4 g/L, *P. italicum* spore was germinated less than 5.0%, whereas a complete inhibitory rate (100%) was attained by the P7G-treated concentration at 0.8 g/L (Table 2).

### 3.3. P7G Inhibits Blue Mold Development on Navel Orange Infected with P. italicum

**Figure 3:**
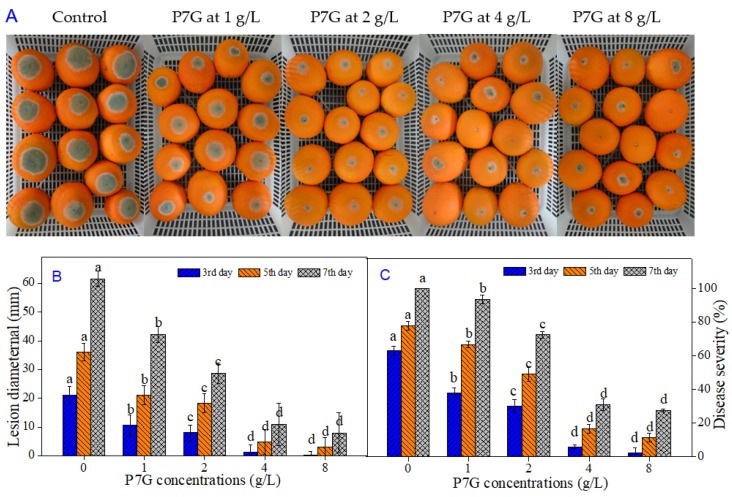
Effects of P7G at concentrations of 1–8 g/L on blue mold disease development of “Newhall” navel oranges wounds inoculated with *P. italicum*. Lesion diameter (**B**) and disease severity (**C**) were measured after 3, 5, and 7 days of incubation at 25 °C. Each column is the mean of three replicates (10 oranges per replicate), and vertical bars represent the standard error (S.E.). The columns with different lowercase letters among each concentration at each time point are significantly different according to Duncan’s test at *p* < 0.05.

To certificate the antifungal efficacy of P7G for controlling postharvest blue mold caused by *P. italicum* in citrus fruit, we implemented an in vivo test on “Newhall” navel oranges. As shown in Figure 3A, blue mold symptoms and disease development in ‘Newhall’ navel orange wounds inoculated with *P. italicum* were significantly (*p <* 0.05) inhibited by P7G in a dose-dependent manner, suggesting a strong antifungal effect against *P. italicum*. The lesion diameter of blue mold infection in P7G-treated fruit at concentrations of 1, 2, 4, and 8 g/L were 50.0%, 62.3%, 94.3%, and 98.4% smaller (*p <* 0.05) than that in the control fruit at day three after inoculation, respectively (Figure 3B). Likewise, P7G treatment with different concentrations significantly reduced disease severity in “Newhall” navel orange wounds inoculated with *P. italicum* (*p* < 0.05). The development of blue mold in “Newhall” navel oranges was significantly affected by P7G in a dose-dependent manner (*p* < 0.05). Among the test concentrations, over five MFC P7G treatments (4 g/L and 8 g/L) showed the greatest inhibitory effect, and had no significant differences in disease severity among the three time points of days three, five and seven after inoculation, respectively (Figure 3C). Based on the above results, the optimal inhibitory concentration of P7G for controlling postharvest blue mold of “Newhall” navel oranges caused by *P. italicum* was 4 g/L.

### 3.4. P7G Alters the Morphology and Ultrastructure of P. italicum

**Figure 4:**
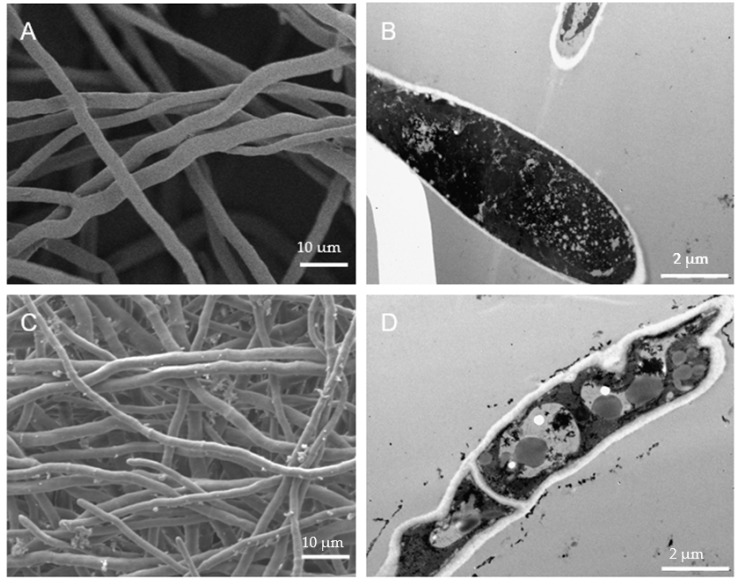

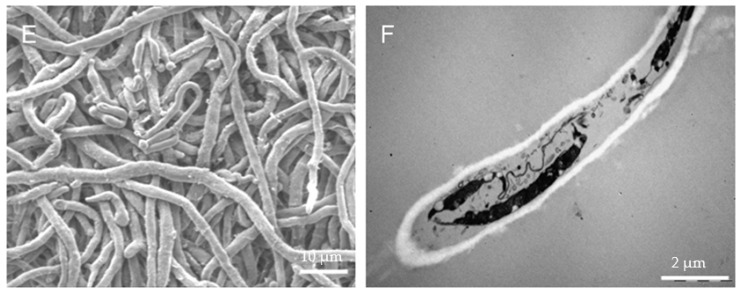
Scanning electron microscopy (SEM) and transmission electron microscopy (TEM) images of P7G against *P. italicum*. (**A**, **B**) Control mycelia and cells; (**C**, **D**) mycelia and cells treated with P7G at EC_50_; (**E**, **F**) mycelia and cells treated with P7G at MIC.

To explore the antifungal mechanism of P7G against *P. italicum*, the morphology and ultrastructure of *P. italicum* mycelia and cells treated with P7G at various concentrations (0, EC_50_ and MIC) were observed by SEM and TEM, respectively. The control mycelia of *P. italicum* grown in PDB showed smooth and homogeneous mycelial morphology (Figure 4A), with a typical fungal ultrastructure, abundant cytoplasmic matrix, and ordered division (Figure 4B). By contrast, these normal mycelial morphology and cell structures were changed conspicuously when exposed to P7G. Those treated with P7G at EC_50_ (0.08 g/L) had an obvious buckling, abnormal enlargement of the growing point, and a rough surface (Figure 4C); meanwhile, the cell membrane collapsed, and degradation of cytoplasmic organelles led to the appearance of large vacuoles (Figure 4D). Furthermore, the mycelia of *P. italicum* treated with P7G at MIC (0.2 g/L) were greatly damaged, and some of them parted and broke (Figure 4E); these destroyed phenomena of plasmolysis, indistinct organelles, thinned cell wall, infrequent cytoplasmic matrix, and cell permeabilization appeared in *P. italicum* cells treated with 0.2 g/L of P7G (Figure 4F).

### 3.5. P7G Causes Cell Membrane Damage of P. italicum

**Figure 5:**
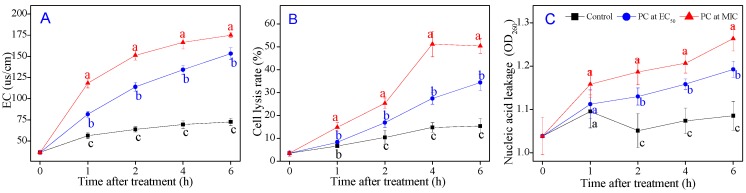
Effects of P7G on extracellular conductivity (**A**), cell lysis rate (**B**) and nucleic acid leakage (**C**) of *P. italicum* in potato dextrose broth (PDB). Each value is the mean of three replicates (*n* = 3), and the vertical bar indicates the standard error (S.E.). The values with different lowercase letters within the same time point indicate a significant difference between the control and the P7G-treated mycelia according to Duncan’s test at *p* < 0.05.

To further investigate the damage of the cell membrane induced by P7G treatment, the extracellular conductivity (EC), cell lysis rate and nucleic leakage of *P. italicum* were tested, all of which have been frequently used for determining the damage of membrane permeability. The EC of *P. italicum* supernatant treated with P7G at different concentrations (0, EC_50_, and MIC) for an inoculation period of 0–6 h was depicted in Figure 5A, and increased constantly with P7G-treated time. The electrical conductivity of 0.08 and 0.2 g/L P7G-treated *P. italicum* supernatant was 153.3 μs/cm and 175.1 μs/cm, respectively after 6 h of P7G treatment, being about 2.11 and 2.42 times higher than that of the control samples (72.5 μs/cm; *p* < 0.05). As demonstrated in Figure 5B, the cell lysis rate in *P. italicum* supernatant treated with P7G exhibited obvious increases compared to the control samples particularly, which reached the highest value after 4 h of P7G treatment and was maintained at higher levels by P7G treatment with MIC (0.2 g/L) than that in EC_50_ (0.08 g/L). Simultaneously, the higher the treatment concentration of P7G, the greater the nucleic acid leakage from the *P. italicum* supernatant; the supernatant with P7G at EC_50_ (0.08 g/L) at 6 h of the incubation had an absorbance at 260 nm (OD_260_) of 1.193, which was significantly higher (*p* < 0.05) than that of the control samples (1.085), but significantly lower than that with MIC of P7G. Nevertheless, the OD_260_ values of *P. italicum* supernatant had no significant difference between the P7G-treated and control samples before 1 h of P7G treatment (Figure 5C). Thus, P7G treatment contributed to increasing the extracellular conductivity, cell lysis rate, and nucleic acid leakage in *P. italicum* supernatant, resulting in membrane permeability damage.

### 3.6. P7G Induces the Outflow of Intercellular Inclusions of P. italicum

**Figure 6:**
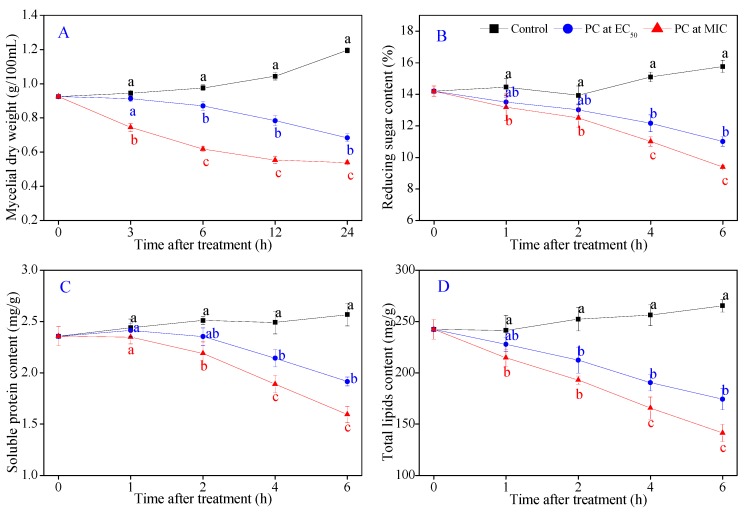

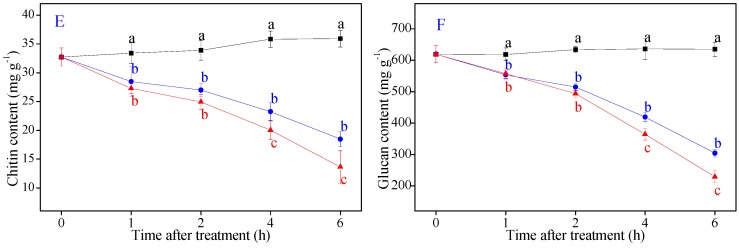
Effects of P7G on mycelial dry weight (**A**), reducing sugar content (**B**), soluble protein content (**C**), total lipid content (**D**), chitin content (**E**) and glucan content (**F**) of *P. italicum* in PDB. Each value is the mean of three replicates (*n* = 3), and the vertical bar indicates the standard error (S.E.). The values with different lowercase letters within the same time point indicate a significant difference between the control and the P7G-treated mycelia, according to Duncan’s test at *p* < 0.05.

Intracellular inclusions, as the indispensable material basis for the growth and reproduction of microorganisms, play a dominant role in biosynthesis, energy metabolism, and signal transduction. The present study evaluated the cell membrane damage of P7G on mycelial growth and intracellular constituent contents of *P. italicum* mycelia. As illustrated in Figure 6A, P7G treatment significantly suppressed the mycelial growth of *P. italicum* in PDB (*p* < 0.05). After 6 h of P7G treatment, the mycelial growth weight of *P. italicum* was 0.870 ± 0.027 g/(100 mL) and 0.618 ± 0.013 g/(100 mL) when the treatment concentration of P7G was 0.08 g/L and 0.2 g/L, which was significantly lower than that in the control samples (0.957 ± 0.017 g/(100 mL); *p* < 0.05). After being re-culture for 12 and 24 h, the mycelial growth weight of *P. italicum* treated with P7G at MIC (0.2 g/L) had no significant difference. As demonstrated in Figure 6B–D, the contents of reducing sugar, soluble protein and total lipid in the control mycelia were found to maintain a steady upward level during the whole inoculation period. As shown in Figure 6B, the reducing sugar content in *P. italicum* mycelia significantly decreased from 15.10 ± 0.31 mg/g without P7G to 19.47% and 27.15% with P7G at EC_50_ (0.08 g/L) and MIC (0.2 g/L), respectively. Moreover, the reducing sugar content in 0.08 and 0.2 mg/mL P7G-treated *P. italicum* mycelia were 11.01 ± 0.30 mg/g and 9.39 ± 0.11 mg/g, respectively after 6 h of P7G treatment, much lower than that in the control samples (15.77 ± 0.39 mg/g). Similarly, the soluble protein content of *P. italicum* mycelia significantly decreased with P7G treatment at EC_50_ and MIC during the entire incubation (Figure 6C). At the end of the inoculation period (6 h), the soluble protein content of the 0.08 g/L and 0.2 g/L P7G-treated *P. italicum* mycelia were 1.92 ± 0.04 mg/g and 1.59 ± 0.08 mg/g, respectively, which were significantly lower than that in the control samples (2.57 ± 0.11 mg/g; *p* < 0.05). The effect of P7G at different concentrations (0, EC_50_, and MIC) on the total lipid contents of *P. italicum* was shown in Figure 6D. The total lipid contents in P7G treatments were significantly lower than that in control samples during the entire incubation. After 6 h of P7G treatment, the total lipid contents in 0.08 and 0.2 g/L P7G-treated *P. italicum* mycelia were 174.3 ± 10.3 mg/g and 141.1± 8.4 mg/g, respectively, which were decreased by 34.30% and 46.81% compared to the control samples (265.7 ± 6.1 mg/g). The above results were highly aligned with the microscopic observations in this study, and indicate that P7G treatment led to the inhibition of the synthesis of intracellular inclusions that caused cell membrane damage.

### 3.7. P7G Modifies the Cell Wall Structure of P. italicum

To further investigate the damage of the cell wall induced by P7G treatment, the cell wall constituent contents and degrading enzyme activities of *P. italicum* mycelia were tested, which are frequently used for determining the damage of the cell wall. As shown in Figure 6E, the chitin content in *P. italicum* mycelia treated with P7G at EC_50_ and MIC significantly declined to 18.48 ± 1.33 mg/g and 13. 63 ± 2.89 mg/g, which were 48.52% and 60.03% lower than that of the control mycelia (*p <* 0.05), respectively. Similarly, a continuous decrease in the glucan content of *P. italicum* after P7G treatment was observed during the entire incubation, whereas the glucan content in control samples remained stable (Figure 6F). After P7G treatment for 6 h, the glucan content in *P. italicum* mycelia treated with EC_50_ and MIC were 304.1 ± 11.8 mg/g and 228.9 ± 19.1 mg/g, respectively, which were significantly lower than that in the control samples (634.9 ± 22.6 mg/g; *p <* 0.05).

**Figure 7:**
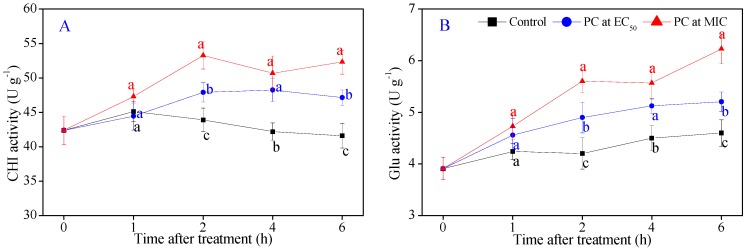
Effects of P7G on chitinase (CHI) activity (**A**) and β-1,3-glucanase (β-Glu) activity (**B**) of *P. italicum* in PDB. Each value is the mean of three replicates (*n* = 3), and the vertical bar indicates the standard error (S.E.). The values with different lowercase letters within the same time point indicate a significant difference between the control and the P7G-treated mycelia, according to Duncan’s test at *p* < 0.05.

As shown in Figure 7A,B, there was almost no change in the CHI and β-Glu activities in the control samples during the entire incubation. As illustrated by Figure 7A, the CHI activity in the control *P. italicum* mycelia began to decrease after 1 h of P7G treatment and remained stable thereafter, whereas the CHI activity of *P. italicum* mycelia treated with P7G at EC_50_ and MIC showed a significant uptrend after 1 h of the treatment. The β-Glu activity significantly increased in the *P. italicum* mycelia treated with EC_50_ and MIC of P7G at respective levels 1.13- and 1.36-fold higher than that in the control samples after 6 h (Figure 7B).

## 4. Discussion

Medicinal plant extracts and their bioactivators are being paid increasing attention by researchers due to their potential to control fungal growth as well as their preservative effects [3,21,25,30,37,38]. Among plant extracts, *Ficus hirta* Vahl. fruit is a common medicine and food that is widely distributed in southern China, and has gained a great deal of attention as an alternative antifungal agent due to its antifungal and antioxidant capacity owing to its high flavonoid content [18,23,31]. In our previous study, we confirmed that P7G isolated from *F. hirta* Vahl. fruit displayed a significant antifungal activity against postharvest pathogens of citrus fruit, especially for *P. italicum* [18,23,29]. To the best of our knowledge, no studies on the antifungal efficacy and possible mechanisms of P7G against this pathogen have yet been reported. In the current study, we showed that P7G had a prominent in vitro inhibitory effect on the mycelial growth, spore germination, mycelium morphology, cell ultrastructure, membrane permeability, intracellular constituents contents and cell wall degrading enzymes activities of *P. italicum*. In addition, P7G treatment showed in vivo antifungal efficacy in reducing postharvest blue mold of citrus fruit.

The in vitro antifungal inhibition of P7G on mycelial growth and spore germination of *P. italicum* was shown to be in a prominent dose-dependent manner. Furthermore, in the in vitro study, our results demonstrated that, in comparison with the control group, the colony diameter of *P. italicum* dropped to 50% when P7G achieved 0.08 g/L on PDA; meanwhile, the germination rate of *P. italicum* spore was suppressed by half in 0.032 g/L P7G in PDB, indicating that in comparison to mycelia, the spore germination of *P. italicum* was more sensitive to P7G treatment (Figure 2 and Table 2). P7G gave lower MIC and MFC values of 0.2 and 0.8 g/mL, respectively, and showed higher antifungal activity against *P. italicum* than that determined for some other plant-derived antifungal active compounds (e.g., α-terpineol and pinocembrin) assayed in previous studies [36,39]. For example, the effective antifungal concentration of the commonly used fungicide imazalil (IMZ) against green and blue molds requires 0.4 g/L [40]. In the in vivo experiments herein, P7G treatment significantly reduced the lesion diameter and disease development of blue mold in “Newhall” navel orange wounds inoculated with *P. italicum*, with a lower disease severity of P7G-treated (5 × and 10 × MFC) fruit of only 30.9 ± 3.7% and 27.4 ± 1.0% after 7 days post inoculation (Figure 3), which is consistent with those previously reported [41,42,43,44]. Recent studies have reported the antifungal potential of P7G in citrus fruit, while the results of the current study showed a strong check on the disease development of blue mold in citrus fruit and in vitro growth of *P. italicum* when exposed to various concentrations of P7G. Therefore, it is suggested that P7G could be a natural alternative for routine synthetic antifungal agents to control the growth of *P. italicum*, which causes postharvest blue mold rot in citrus fruit and its related horticultural products [45,46].

This is a neoteric study in which P7G treatment was shown to have an effective in vivo control efficacy and in vitro inhibitory effect. Thus, the antifungal mechanisms of P7G deserve to be further elucidated. In general, flavonoids can inhibit the growth of phytopathogens, particularly fungal pathogens, mainly by damaging the cell membranes [47]. Numerous reports have elucidated the antifungal properties of natural antifungal compounds sourced from different plant extracts or essential oils that are largely due to the damage of the cell membrane [28,33,48]. In our study, the SEM images showed that P7G notably caused the formation of a rough surface and irregular shrinkage of *P. italicum* mycelia (Figure 4). These findings were also confirmed by the results of TEM analysis of *P. italicum* cells in which plasmolysis, indistinct organelles, outflowed cytoplasmic matrixces, thinned cell walls, and cavitation were observed in P7G-treated cells (Figure 4D,F). The observed changes in P7G-treated mycelia and cells may be due to an increase in cell permeabilization. Thus, we inferred that the in vitro antifungal mechanism of P7G against *P. italicum* may be associated with the dissociation of the fungal cell membranes.

Fungal cell membranes have a vital function in maintaining the relative stability of the cell cytoplasm, and previous studies have reported that various natural plant-derived compounds are potential fungicides and fungistats that directly or indirectly target the cell membrane or its components to disrupt membrane permeability, to destroy the membrane structure, and to cause leakage of nucleic acids [28,32,33]. Membrane permeability parameters, including changes in extracellular conductivity, cell lysis rate, and the leakage of nucleic acids, were used to verify this phenomenon. Sugars, proteins and lipids are the primary components of cell membranes, and the loss of cell membrane components can typically indicate irreversible damage to the cell membrane [3,32,49]. In the present study, P7G treatment led to a rapid, significant increase in the extracellular conductivity, cell lysis rate, and nucleic acid leakage in *P. italicum* supernatants (Figure 5), as well as to a significant decrease in mycelial weight and in reducing sugar, soluble protein, and total lipid contents in *P. italicum* mycelia (Figure 6). Similar findings describing the inhibitory effects of cinnamaldehyde, citral and pinocembrin on *P. italicum* growth have been reported [32,33,36]. These findings indicate that membrane stability is an important antifungal target of P7G.

The cell wall is a specific organelle of fungi that plays an important role in maintaining the inherent form, integrity, and normal metabolism of fungal cells. Chitin and glucan are two of the primary components of fungal cell walls and are important indicators that reflect the growing states of phytopathogens [50]. Both CHI and β-Glu are considered to be two key cell wall-degrading enzymes that openly inhibit the growth of pathogens primarily by the decomposition of chitin and glucan in the cell wall of many pathogenic fungi [51]. In our study, both the chitin and glucan contents of *P. italicum* mycelia were significantly inhibited by P7G at EC_50_ and MIC (Figure 6E,F), suggesting that P7G treatment could significantly suppress the biosynthesis of chitin and glucan, which is highly consistent with a recent report by Ouyang et al. [52]. Moreover, P7G treatment significantly increased the activities of the two cell wall-degrading enzymes CHI and β-Glu in *P. italicum* mycelia compared to that observed in the control mycelia (Figure 7A,B). These results support the observed ultrastructure of *P. italicum* cells treated with P7G, which is in high agreement with a previous report by Li et al. [53]. Taken together, the observed decrease in cell wall contents and the increase in cell wall-degrading enzyme activities demonstrate that the fungal cell wall is a crucial antifungal target of P7G.

## 5. Conclusions

In summary, this study demonstrated that P7G is effective at inhibiting the in vitro mycelial growth of *P. italicum*, and hence controls the postharvest blue mold on citrus fruit. The SEM and TEM observations showed that P7G treatment damaged the morphology of *P. italicum* mycelia by increasing the leakage of cytoplasmic constituents, altering membrane permeability and destroying cell wall structure, resulting in *P. italicum* cell death. Taken together, the antifungal potential of P7G may be attributed to causing significant damage to the structures of fungal cell membrane, as well as cell wall. These results imply that P7G has the antifungal potential to be a promising novel botanical fungicide to control blue mold disease in citrus fruit; however, further studies are needed to assess its ability to promote “Newhall” navel orange preservation.

## Figures and Tables

**Table 1:** Determination of minimal inhibitory concentration (MIC) and minimum fungicidal concentration (MFC) values of pinocembrin-7-glucoside (P7G) against postharvest pathogenic fungi in citrus fruit.

Concentration of P7G (g/L)		Pathogenic Fungus					
		*Alternaria citri*	*Colletotrichum gloeosporioides*	*Diaporthe citri*	*Geotrichum citri-aurantii*	*Penicillium* digitatum	*Penicillium italicum*
Mycelial growth for 2nd d	0	+	+	+	+	+	+
	0.025	+	+	+	+	+	+
	0.05	+	+	+	+	+	+
	0.1	+	+	−	+	+	+
	0.2	+	−	−	−	−	−
	0.4	+	−	−	−	−	−
	0.8	−	−	−	−	−	−
Mycelial growth for 6th d	0	+	+	+	+	+	+
	0.025	+	+	+	+	+	+
	0.05	+	+	+	+	+	+
	0.1	+	+	+	+	+	+
	0.2	+	+	+	+	+	+
	0.4	+	−	−	+	−	+
	0.8	−	−	−	−	−	−
**MIC**		0.8	0.2	0.1	0.2	0.2	0.2
**MFC**		0.8	0.4	0.4	0.8	0.4	0.8

Note: “+”, presence of mycelia growth; “−”, absence of mycelia growth.

**Table 2:** Inhibitory effect of P7G on spore germination of *P. italicum*.

P7G Concentration (mg/mL)	Spore Germination (%)	Inhibitory Rate (%)	EC_50_ (mg/mL)
0	93.40 ± 1.90 a	0.00 ± 0.00 g	0.032
0.025	67.17 ± 2.05 b	28.09 ± 2.20 f	
0.05	46.50 ± 1.60 c	50.21 ± 1.71 e	
0.1	24.60 ± 1.20 d	73.66 ± 1.28 d	
0.2	13.92 ± 0.87 e	85.10 ± 0.94 c	
0.4	3.50 ± 0.20 f	96.25 ± 0.21 b	
0.8	0.00 ± 0.00 g	100.00 ± 0.00 a	

Data represent the mean ± standard error (S.E.; *n* = 4). Different letters (a–g) indicate significant differences at *p* < 0.05 according to Duncan’s test.
